# Development and application of an interaction network ontology for literature mining of vaccine-associated gene-gene interactions

**DOI:** 10.1186/2041-1480-6-2

**Published:** 2015-01-06

**Authors:** Junguk Hur, Arzucan Özgür, Zuoshuang Xiang, Yongqun He

**Affiliations:** Department of Neurology, University of Michigan, Ann Arbor, MI 48109 USA; Department of Computer Engineering, Bogazici University, 34342 Istanbul, Turkey; Unit for Laboratory Animal Medicine, University of Michigan, Ann Arbor, MI 48109 USA; Department of Microbiology and Immunology, University of Michigan, Ann Arbor, MI 48109 USA; Center for Computational Medicine and Bioinformatics, University of Michigan, Ann Arbor, MI 48109 USA; Comprehensive Cancer Center, University of Michigan, Ann Arbor, MI 48109 USA

**Keywords:** Biomedical ontology, Interaction network ontology, Literature mining, Interaction enrichment, Gene-gene interaction

## Abstract

**Background:**

Literature mining of gene-gene interactions has been enhanced by ontology-based name classifications. However, in biomedical literature mining, interaction keywords have not been carefully studied and used beyond a collection of keywords.

**Methods:**

In this study, we report the development of a new Interaction Network Ontology (INO) that classifies >800 interaction keywords and incorporates interaction terms from the PSI Molecular Interactions (PSI-MI) and Gene Ontology (GO). Using INO-based literature mining results, a modified Fisher’s exact test was established to analyze significantly over- and under-represented enriched gene-gene interaction types within a specific area. Such a strategy was applied to study the vaccine-mediated gene-gene interactions using all PubMed abstracts. The Vaccine Ontology (VO) and INO were used to support the retrieval of vaccine terms and interaction keywords from the literature.

**Results:**

INO is aligned with the Basic Formal Ontology (BFO) and imports terms from 10 other existing ontologies. Current INO includes 540 terms. In terms of interaction-related terms, INO imports and aligns PSI-MI and GO interaction terms and includes over 100 newly generated ontology terms with ‘INO_’ prefix. A new annotation property, ‘has literature mining keywords’, was generated to allow the listing of different keywords mapping to the interaction types in INO. Using all PubMed documents published as of 12/31/2013, approximately 266,000 vaccine-associated documents were identified, and a total of 6,116 gene-pairs were associated with at least one INO term. Out of 78 INO interaction terms associated with at least five gene-pairs of the vaccine-associated sub-network, 14 terms were significantly over-represented (*i.e.*, more frequently used) and 17 under-represented based on our modified Fisher’s exact test. These over-represented and under-represented terms share some common top-level terms but are distinct at the bottom levels of the INO hierarchy. The analysis of these interaction types and their associated gene-gene pairs uncovered many scientific insights.

**Conclusions:**

INO provides a novel approach for defining hierarchical interaction types and related keywords for literature mining. The ontology-based literature mining, in combination with an INO-based statistical interaction enrichment test, provides a new platform for efficient mining and analysis of topic-specific gene interaction networks.

## Background

Two common strategies of literature retrieval of reported gene-gene interactions include gene-gene co-occurrence and interaction keywords-based literature mining. In this paper, the gene-gene interaction represents a broad interactive relation between two genes or gene products [1]. Such a relation does not have to be a direct physical interaction. The co-occurrence strategy identifies two related genes both listed in the same literature, or more specifically in the same title, abstract, or sentence. An example of such a strategy is PubGene, which extracts gene relationships based on the co-occurrence of gene symbols in MEDLINE titles and abstracts [2]. The other strategy relies on the identification of two genes together with an interaction keyword in the same sentence. Such a method may still generate many false-positive results. To improve the interaction keyword-based approach, machine learning algorithms (e.g., support vector machine (SVM) [3]) with features extracted from syntactic analysis of sentences (e.g., dependency parse trees) can be used [4].

Ontologies can be applied to enhance literature mining performance. For example, in our previous work, a vaccine-specific sub-network was built by considering only the interactions that were extracted from sentences that contain the “vaccine” term (or its variants like “vaccines”, “vaccination”, and “vaccinated”). This strategy does not retrieve the sentences where more specific vaccine names such as BCG (a commercial tuberculosis vaccine) are mentioned. Such vaccine names and their hierarchical relations are represented in Vaccine Ontology (VO) [5]. We found that the application of VO has significantly improved the analysis of the vaccine-specific sub-networks [6].

An ontology that logically represents various interaction keywords/types and their semantic relations would help address the challenge of retrieving and classifying the types of gene-gene interactions in the interaction keyword-based literature mining. The GENIA ontology provides a semantically annotated corpus for biological literature mining [7]. However, this ontology does not specify various types of interactions between genes or proteins. Initiated from the classification of >800 interaction keywords [6], we have developed the Interaction Network Ontology (INO) that ontologically represents various interaction types and their relations, and collects and assigns interaction keywords to these different interaction types. The details about the ontology will, for the first time, be provided in this manuscript.

In addition to supporting the literature mining of gene-gene interactions, INO can be used for interaction type enrichment analysis. Gene Ontology (GO)-based gene set enrichment analyses have been widely used to determine over- or under-represented biological functions in a set of genes obtained from high-throughput Omics studies. GO provides controlled vocabulary of standard terms for describing gene product characteristics in a hierarchical structure. The input to the GO term enrichment analysis is a list of genes. Such a method does not classify enriched gene-gene interactions. Since INO classifies different interaction types into a structured ontology, it becomes possible to perform a gene-gene interaction enrichment study by comparing the INO-based literature-mined data of gene-gene interactions in some specific domain over the data from the broad background.

In this manuscript, we will first introduce the development of INO with a focus on its representation of interaction types and keywords for literature mining. An INO-based gene interaction enrichment method based on a modified Fisher’s exact test will then be introduced. We applied our approach to the analysis of the vaccine-mediated gene-gene interactions. The resulting over- and under-represented gene-gene interaction types and gene-gene interactions will also be described in detail.

## Methods

### INO development

INO was developed by following the Open Biological Ontology (OBO) Foundry ontology development principles, including openness and collaboration [8]. Its development is aligned and integrated with existing OBO Foundry library ontologies. INO imports existing terms by using OntoFox [9]. New terms generated in INO use the “INO_” prefix. INO uses the format of W3C standard Web Ontology Language (OWL2) (http://www.w3.org/TR/owl-guide/). For efficient editing of INO, the Protégé 4.3 OWL ontology editor (http://protege.stanford.edu/) was used.

The INO source is open freely under a Creative Commons (CC) license for public and commercial usage. INO has been deposited at the INO SourceForge project page (http://sourceforge.net/projects/ino/). It is also available in the ontology repositories of National Center for Biomedical Ontology (NCBO) BioPortal (http://purl.bioontology.org/ontology/INO) and Ontobee [10] (http://www.ontobee.org/browser/index.php?o=INO).

### INO-based literature mining of gene-gene interaction pairs and interaction types

The sentences from the complete PubMed abstracts (published up to 12/31/2013) were obtained from the BioNLP database in the National Center for Integrative Biomedical Informatics (http://ncibi.org/). Our in-house literature mining tools, SciMiner [11] and VO-SciMiner [12], were used to identify gene names/symbols and VO and INO terms (interaction keywords) from these sentences. Sentences with two gene names and at least one INO term (e.g., interacts, binds, activates) were selected. We obtained the dependency parse trees of the sentences using the Stanford Parser [13] and extracted the shortest dependency path between each pair of genes in a sentence. We defined an edit distance-based kernel function among these dependency paths and used SVM [3] to classify whether a path describes an interaction between a gene pair [6]. A confidence score calculated based on SVM was used to measure the confidence of association between two genes in a sentence in the literature. Positively-scored sentences were kept, and the gene pairs together with the interaction keywords from these sentences were extracted. The extracted interaction keywords were mapped to INO to define the interaction types.

### Development of INO-based statistical enrichment analysis of literature mined gene-gene interaction data

A modified Fisher’s exact test has gained popularity over the last decade in high-throughput gene expression studies as a preferred method for identifying enriched biological functions among given gene sets [14, 15]. We implemented the modified Fisher’s exact test in Perl using the Ngram Statistics Package [16] to identify enriched gene-gene interaction types, in terms of INO terms, within a concept-specific sub-network. For each INO term, a 2×2 contingency table is obtained on which the Fisher’s test runs, as shown in Table 1. Both significantly under-represented and over-represented terms are selected as a significantly enriched INO term with a p-value < 0.05 after Benjamini-Hochberg (BH) multiple testing corrections. Here a significantly over-represented or under-represented term indicates that the term was significantly more or less frequently used in the vaccine context compared to the whole literature background. In the current study, a vaccine-associated gene-gene interaction network was defined based on the gene-gene interactions obtained from the PubMed abstracts, including those retrieved by a PubMed search of ‘vaccine’ and those identified by VO-SciMiner using 186 specific vaccine terms extracted from the VO ‘vaccine’ branch. These 186 vaccine terms (e.g., tuberculosis vaccine BCG) are easily identified by natural language processing programs. This vaccine-associated network was compared against the complete gene-gene interaction network.

**Table 1:** **The 2x2 contingency table**

# of gene-gene	Concept-	Whole
Interaction pairs	specific sub-network	Network
With the INO term	30 – 1	500
Without the INO term	150	30000

Note: The sub-network has 30 gene pairs associated with this INO term out of a total of 180 gene pairs. A modified Fisher’s exact test, with the “- 1” modification made to the typical Fisher’s exact test to make the statistical test more conservative, was employed to identify significantly over-represented terms (p-value of 6.9E-20).

## Results

### The Interaction Network Ontology (INO)

#### (1) INO overall design and hierarchy

**Figure 1:**
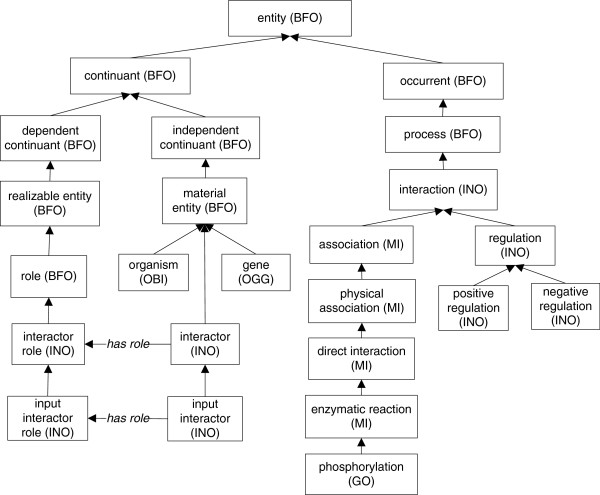
**INO hierarchy and selected INO key terms.** INO is aligned with BFO. It imports most PSI-MI interaction type terms to represent the various interaction types. Some bottom level interaction terms (*e.g.*, phosphorylation) are replaced with corresponding GO terms. Many INO-specific terms (*e.g.*, regulation) that do not exist in PSI_MI or GO are also generated. Note that there are different interactors but only input interactor is shown here. The network and pathway related terms are not shown.

INO is a biomedical ontology in the domain of molecular interactions and interaction networks. INO is aligned with the upper-level Basic Formal Ontology (BFO) [17] (Figure 1). BFO contains two branches, continuant and occurrent. The continuant branch represents time-independent entities such as material entity, and the occurrent branch represents time-related entities such as process. BFO has currently been used by over 100 domain ontologies, including many (*e.g.*, GO) within the framework of the OBO Foundry [8]. By aligning different domain ontologies under the two branches of BFO, INO is able to efficiently use the terms from other ontologies in representing signaling pathway elements.

Three important INO terms are interaction, network, and pathway. In INO, an interaction is defined as a processual entity that has two or more participants (i.e., interactors) that have an effect upon one another under a particular condition. An interactor (or called interactant) is defined as a material entity that plays the role of “interactor role”. With different roles, an interactor can be an ‘input interactor’, ‘output interactor’, ‘catalyst’, ‘positive regulator’, or ‘negative regulator’. An interaction consumes its input interactors (but not the catalysts or regulators) and generates its output interactors. A network is a process that includes at least two connected interactions. A network does not have to include a predefined start or end entity. A pathway is a type of network that has specified distinct start(s) and end(s). Each of these three INO terms includes many subclasses. Therefore, in addition to the representation of various interaction types, INO has also been developed to represent pathways and networks. Furthermore, INO has been used as a species-neutral ontology core and platform for generating human-specific interaction network ontology (HINO) [18, 19]. Since the scope of this manuscript is the ontology-based literature mining of gene-gene interactions, we will primarily focus on the ontological representation of interactions in INO.

**Figure 2:**
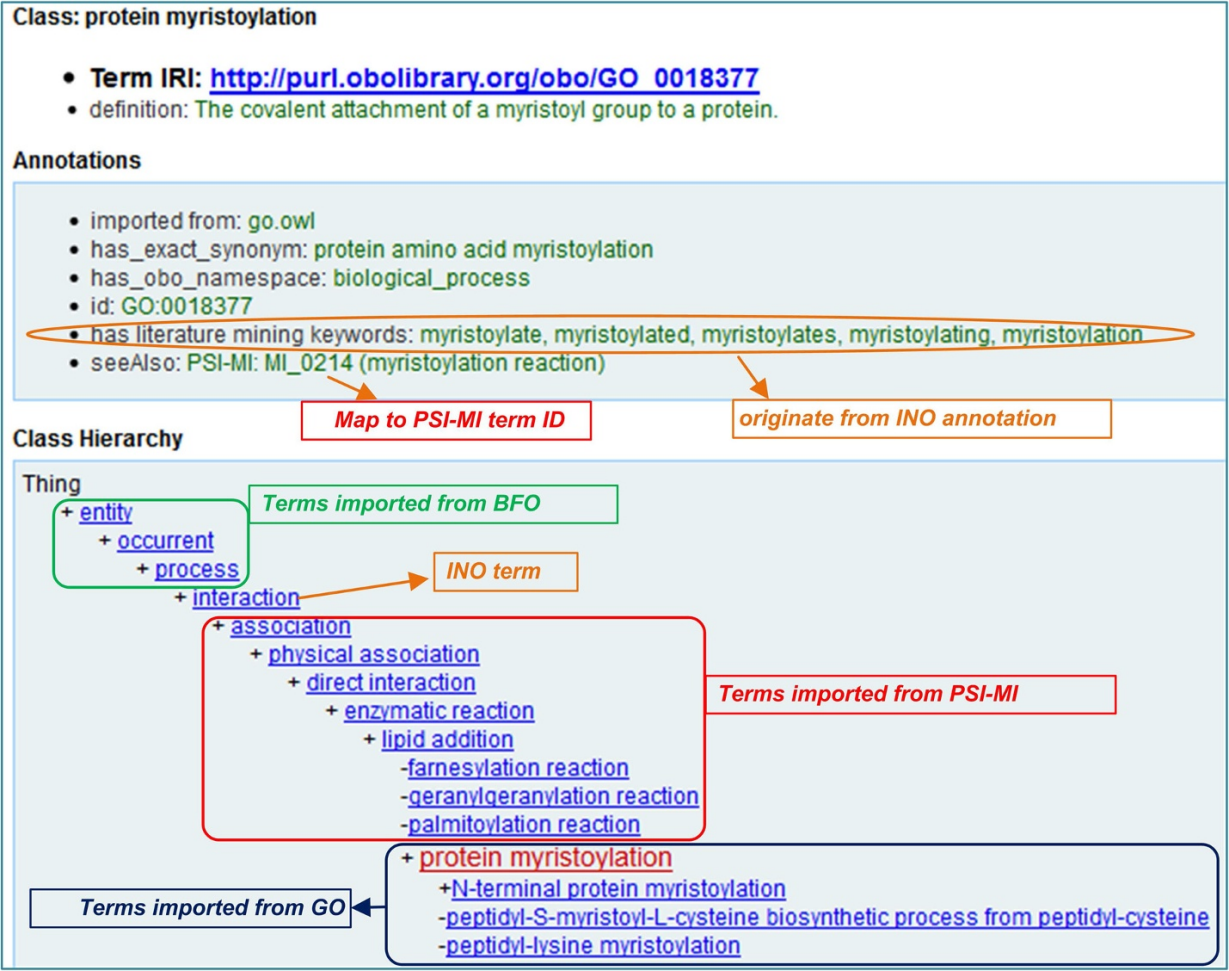
**The visualization of one term ‘protein myristoylation’ (GO_0018377) in INO.** Originated from GO, this term and its branch of child terms are imported and placed with the framework of PSI-MI interaction types which are also imported into INO. The upper level terms are from BFO. The OntoFox tool [9] was used for importing external ontology terms and their axioms. The image is a screenshot generated from Ontobee [10]. To facilitate literature mining tagging, different synonyms of the term are collected under an annotation note.

INO imports terms from other ontologies, particularly from the Proteomics Standard Initiative-Molecular Interaction (PSI-MI), which is a standard molecular interaction data exchange format established by the Human Proteome Organization (HUPO) Proteomics Standard Initiative (http://www.psidev.info). Their PSI-MI format has been widely used in the proteomics community and PSI-MI is also an OBO Foundry library ontology. To be compatible with PSI-MI, we have imported the branch of the ‘interaction type’ (MI_0190) to INO (Figures 1 and 2).

Compared to PSI-MI, GO Biological Processes (BP) branch often has more detailed subclasses (or subtypes) to specific interaction types. Using more general PSI-MI terms (e.g., PSI-MI ‘lipid addition’) as parent terms, INO has imported many specific GO subtypes of interactions (*e.g.*, GO ‘protein myristoylation’) to INO as subclasses of the MI-based interaction terms (Figure 1). As a specific example, we have imported GO ‘protein myristoylation’ and all of its GO subclasses to INO (Figure 2). The GO term ‘protein myristoylation’ has been used to replace the PSI-MI term ‘myristoylation reaction’. It is noted that the top level GO Biological Processes hierarchy is not used because many biological processes (*e.g.*, ‘metabolic process’) in GO are not ‘interaction’ per se and thus cannot be imported to INO for interaction representation.

While PSI-MI focuses on direct protein-protein interactions, it does not include many other interaction types such as regulation types. Therefore, INO also includes interaction terms that are out of current PSI-MI scope, especially different regulation types (Figure 1). Many of these interaction types were generated by classifying the over 800 interaction keywords used in our previous literature mining studies [1, 6].

#### (2) Literature mining support in INO

The over 800 interaction keywords used in our previous literature mining studies [1, 6] do not correspond to the same number of interaction types. While an interaction type or term in INO has its ontology ID, such a term may be associated with different synonyms or related keywords that can be used for literature mining. To support identification of genetic interactions in literature, synonyms and related keywords are needed. To meet this need, we have generated an annotation property called ‘has literature mining keywords’ (Figure 2), which allows the listing of different keywords mapping to the interaction type.

For example, the term ‘protein myristoylation’ in INO has five related literature mining terms including ‘myristoylate’, ‘myristoylates’, ‘myristoylated’, ‘myristoylating’, and ‘myristoylation’. These term variations are listed as an annotation of the interaction type using the annotation property ‘has literature mining keywords’ (Figure 2). The list of keywords can be easily extracted from the ontology by SPARQL or other methods and used for literature mining.

#### (3) Statistics of INO terms and interaction keywords

As of October 2014, INO contains 540 terms, including 123 new INO terms and 317 terms imported from 11 existing ontologies. In addition to the aforementioned ontologies, INO also has imported terms from other authoritative domain ontologies such as the Chemical Entities of Biological Interest (ChEBI) [20] and the Ontology of Genes and Genomes (OGG) [21]. Provenance and source ontology IDs are kept in our term importing [9]. The detailed INO term statistics can be found on the Ontobee INO statistics website (http://www.ontobee.org/ontostat.php?ontology=INO).

Particularly, under the branch of INO *interaction*, INO includes a total of 355 terms. In addition, approximately 700 keywords are defined using the annotation property ‘has literature mining keywords’. These INO interaction terms and their associated literature mining keywords can be used for efficient literature text tagging and retrieval of sentences containing these keywords. The usage of these terms and keywords in our literature mining study is described below.

### INO-based literature mining of gene-gene interactions

#### (1) Workflow and system design

**Figure 3:**
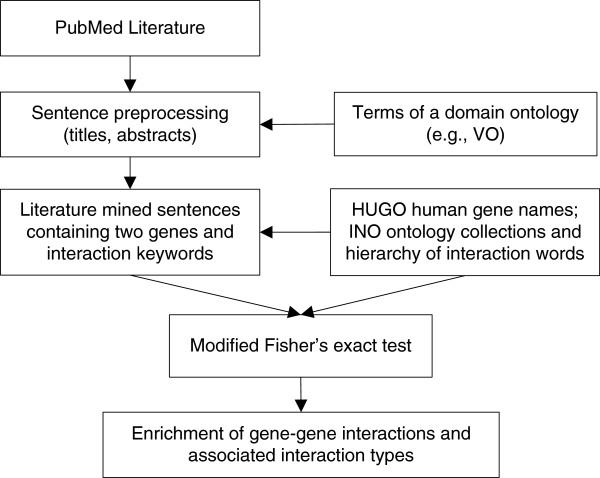
**The workflow of INO-based gene-gene interaction enrichment analysis.** This workflow illustrates the overall procedures of ontology-based gene pair enrichment analysis.

The workflow of the ontology-based gene pair enrichment analysis is illustrated in Figure 3. Specifically, all publications from PubMed were first downloaded. The sentences of article titles and abstracts were parsed and pre-processed. Human gene names and interaction keywords were tagged. To tag human gene names, the HUGO human gene nomenclature assignments (http://www.genenames.org/) were used. These human gene names are also available in the OGG [21]. The INO interaction types and associated keywords were used for tagging interaction keywords. As detailed in the Methods section, an INO-based modified Fisher’s exact test was developed to identify statistically significantly enriched gene-gene interaction types and associated gene-gene pairs (Figure 3).

The INO-based workflow for literature mining of gene-gene interactions is applicable for different use case studies. Below we introduce the application of such a strategy for studying the gene-gene interactions in the vaccine domain.

#### (2) INO-based literature enrichment analysis of vaccine-associated gene-gene interaction data

Our literature mining analysis used all PubMed documents published as of 12/31/2013. A total of 23,481,042 PubMed documents were used as the background data set in the analysis. Using this data set, SciMiner identified 314,152 gene pairs, each of which was associated with at least one INO term.

We applied our study to the vaccine domain. A PubMed search for vaccine-related documents resulted in 237,061 hits (as of 12/31/2013). VO-SciMiner additionally identified 28,908 documents using VO terms, resulting in a total of 265,969 documents to define the vaccine-associated document sets. The gene-gene interactions (*i.e.*, gene pairs) with positive SVM scores and at least one INO term at the same sentence level were compiled from these 265,969 PubMed abstracts. A total of 6,116 gene pairs were associated with at least one INO term.

Out of 78 INO interaction terms associated with at least five gene-pairs of the vaccine-associated sub-network, 14 terms were significantly over-represented (Benjamini-Hochberg (BH) p-value < 0.05 and a minimal enrichment fold of 2) (Table 2). The results indicate that these 14 interaction types are more extensively studied in the vaccine context among the research of all the gene-gene interaction types published in PubMed.

**Table 2:** **Significantly over-represented INO terms among the gene-gene interaction pairs of vaccine-associated sub-network**

INO_ID	Reference term	Enrichment fold	BH * P-value	Most frequent gene-pair (#)
INO_0000140	Neutralization	6.6	0	IFNG_IL12A (5)**
INO_0000096	induction of production	6.2	0	TNF_IFNG (2)
INO_0000106	gene fusion	5.6	0	CD40LG_CD40 (3)
INO_0000103	accessory regulation	3.9	0	CD8A_CD4 (55)
INO_0000062	Costimulation	3.7	0	CD40_CD8A (4)
INO_0000169	Synergization	3.0	0	CD8A_CD40 (5)
INO_0000089	co-regulation	2.9	0	CD8A_CD40 (5)
MI_0559	glycosylation reaction	2.9	0	IL17A_MUC6 (1)
MI_0195	covalent binding	2.5	0	CSF2_ACPP (2)
MI_0208	genetic interaction	4.9	1.82E-10	CD40LG_CD40 (3)
MI_0571	mRNA cleavage	23.2	2.58E-07	CFI_SUPT5H (1)
MI_0902	RNA cleavage	16.2	2.21E-06	CFI_SUPT5H (1)
MI_0910	nucleic acid cleavage	6.4	6.11E-04	CFI_SUPT5H (1)
GO_0018377	protein myristoylation	2.3	2.68E-03	CD4_S100B (2)

*BH: Benjamini-Hochberg; **IFNG_IL12A (5): represents the IFNG and IL12A gene pair with the ‘neutralization’ interaction keyword in five papers.

Furthermore, our gene-gene interaction enrichment analysis was able to retrieve all the gene pairs associated with each interaction type (last column in Table 2). For example, as indicated in five publications (PubMed IDs: 19915058, 8557339, 15557182, 17517055, and 7525727), the cytokines interferon-gamma (IFNG) and interleukin-12A (IL12A) have been found to be closely related, and the neutralization of one cytokine often leads to decreased production of another one [22, 23]. Such *neutralization*-related research is typically found in the field of vaccinology. In another example, associated with the interaction type “induction of production”, the production of one cytokine, TNF (or IFNG), was found to be induced by another cytokine, IFNG (or TNF) [24]. A close examination of all the gene pairs recorded in Table 2 shows that they are all related to the vaccine and immunology research. These results also confirm the specificity of our INO-based enrichment analysis.

In addition, our study found 17 significantly under-represented INO terms with a maximum enrichment fold of 0.5 (equivalent to 2 fold in over-representation) and BH P-value < 0.05 (Table 3). Compared to the general gene-gene interaction research, these interaction types are likely less studied in the vaccinology research field. The reasons of these under-represented interaction types may vary. It is likely that some of these under-represented interactions represent new research opportunities in the vaccinology domain.

**Table 3:** **Significantly under-represented INO terms among the gene-gene interaction pairs of vaccine-associated sub-network**

INO_ID	Reference term	Enrichment fold	BH* P-value
MI_0203	dephosphorylation reaction	0.06	0
INO_0000178	tyrosine-phosphorylation	0.09	0
INO_0000044	gene expression regulation	0.26	0
INO_0000172	transactivation	0.26	0
INO_0000060	coprecipitation	0.28	0
GO_0016310	phosphorylation	0.36	0
MI_0403	colocalization	0.36	0
MI_0414	enzymatic reaction	0.42	0
MI_0194	cleavage reaction	0.49	0
MI_0213	methylation reaction	0.37	6.84E-16
INO_0000092	dissociation	0.28	6.27E-15
INO_0000048	coimmunoprecipitation	0.35	1.00E-13
INO_0000115	hyperphosphorylation	0.27	2.54E-08
INO_0000084	destabilization	0.28	1.49E-05
GO_0006461	protein complex assembly	0.24	1.97E-05
INO_0000088	protein dimerization	0.26	6.41E-05
INO_0000171	Termination	0.42	3.98E-03

*BH: Benjamini-Hochberg.

**Figure 4:**
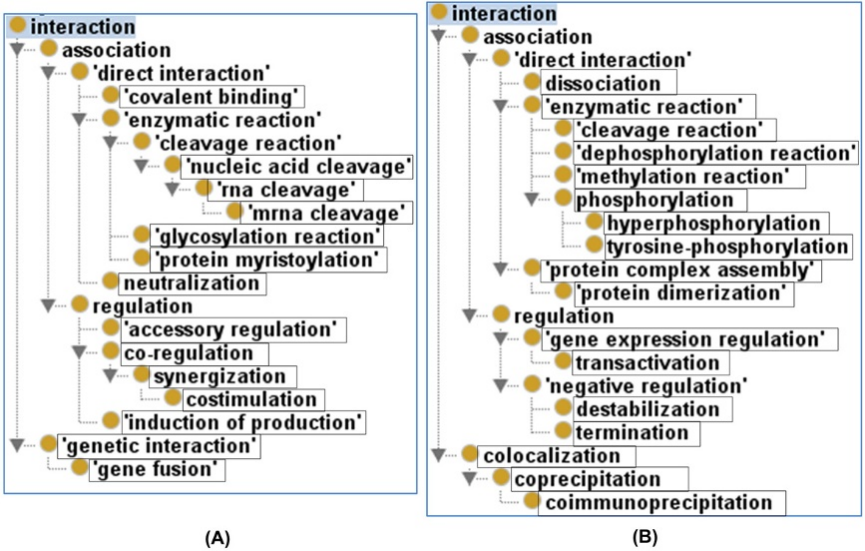
**The hierarchies of over- and under-represented INO interaction terms. (A)** The hierarchy of 14 over-represented INO interaction terms. **(B)** The hierarchy of 17 under-represented INO interaction terms. The results were generated using OntoFox [9] with the OntoFox setting “includeComputedIntermediates”, and visualized using the Protege-OWL editor (http://protege.stanford.edu/). The box-enclosed terms are over- or under-represented interaction types directly identified in our program (see Tables 2 and 3). Other terms not enclosed in boxes are terms retrieved by OntoFox to ensure the completeness of the hierarchies.

One advantage of INO-based study is that we can rely on the INO hierarchy to identify the relations among enriched interaction types. Such a strategy is used to generate the hierarchies of enriched 14 over-represented and 17 under-represented INO interaction types (Figure 4). This study clearly shows the relations between many different interaction terms. For example, among the three over-represented terms, ‘mRNA cleavage’, ‘RNA cleavage’, and ‘nucleic acid cleavage’, there are two parent–child relations as clearly shown in Figure 4. Interestingly, the term ‘cleavage reaction’ is one of the 17 under-represented terms (Table 3). It is noted that the more general term ‘cleavage reaction’ is the parent term of ‘nucleic acid cleavage’, which is the parent term of ‘RNA cleavage’ (Figure 4). The term ‘RNA cleavage’ has a child term ‘mRNA cleavage’. Besides these cleavage types, there are many other specific ‘cleavage reaction’ types, for example, protein cleavage, DNA cleavage, and lipid cleavage. In our calculation of the parent term ‘cleavage reaction’, we included all its child terms. Therefore, the under-represented ‘cleavage reaction’ indicates that the whole category of cleavage reaction is under-represented although the above three specific reaction types are over-represented.

Both sets of over-represented and under-represented interaction terms share some common top-level terms including ‘regulation’, ‘direct interaction’, ‘association’, and ‘interaction’. Otherwise, specific profiles of the two sets are in general distinct at the bottom levels (Figure 4).

## Discussion

This paper introduces two major contributions in the area of ontology-based literature mining research. First, we have for the first time systematically introduced the development of the INO ontology targeting for robust literature mining of gene-gene interaction types. It is noted that in addition to literature mining, INO is also being developed to model various interactions and networks among different molecules [18]. However, the INO development was initiated from meeting our literature mining need [6]. Second, we have proposed and implemented a novel INO-based gene-gene interaction enrichment strategy. The INO-based gene pair enrichment analysis is novel in that the input of such analysis is the literature mined gene-gene interaction types and gene pairs. It differs from a typical GO enrichment analysis where a list of genes is the input. Such a strategy was further used to study the enriched gene-gene interaction types and gene pairs in the domain of vaccinology. Our results demonstrate that the INO offers a repository of hierarchical interaction keywords and a semantic platform for allowing systematical retrieval of interaction types from the literature. The INO-based gene-gene interaction enrichment method further provides a strategy for analyzing the retrieved gene-gene interaction literature mining results.

The coverage of the terms in INO for interaction keywords in literature is wide and includes three sources: (1) The Molecular Interactions (MI) ontology: INO has imported all the interaction-related terms in MI; (2) The Gene Ontology (GO): Many interaction-related GO terms have been imported to INO and aligned with the MI terms; and (3) Newly generated interaction terms in INO: These new interaction-related terms are not available in MI or GO, and thus we generated them in INO with the “INO_” prefix. Furthermore, INO has included many keywords that can be used for literature mining. These literature mining-related keywords are often variations and synonyms of the ontology term labels. The inclusion of these keywords significantly increases our coverage in literature mining. To better understand the interaction term coverage of INO, we have compared the INO system with the commonly used GENIA terminology system [7]. The GENIA term annotation system is grounded on the GENIA ontology that defines biomedically meaningful nominal concepts. Our comparison found that INO covers all 17 interaction types in the GENIA ontology.

To further examine the interaction term coverage of INO, we have also compared our system with the interaction terminology collection from the BioNLP Shared Task 2009, focusing on recognition of bio-molecular events reported in the biomedical literature (http://www.nactem.ac.uk/tsujii/GENIA/SharedTask/). Nine categories of bio-events were covered: gene expression, transcription, protein catabolism, localization, binding, phosphorylation, regulation, positive regulation, and negative regulation [25]. We used the BioNLP’09 Shared Task training data set that consists of 800 abstracts manually labeled for bio-molecular events including the event trigger words (i.e., interaction keywords). These abstracts include 994 unique interaction keywords that are shown for 6,607 times in the data set. Our comparative analysis found that INO includes 279 of these 994 unique interaction keywords. These 279 keywords are used for 4,448 times, which corresponds to 67% of coverage if the keyword redundancy is considered. It is noted that many keywords (e.g., by, when, source, products, necessary, through) listed in the BioNLP’09 Shared Task training data are not considered as interaction keywords in INO. We will fully examine all the terms in the BioNLP’09 Shared Task data set and hopefully expand INO to include more interaction keywords.

Our INO-based literature mining study found that while it is relatively easy to describe the relation between two genes when only one interaction keyword exists in the sentence containing these two genes, it is difficult to describe the relation between the two genes if multiple keywords exist. For example, in the IFNG-IL12A neutralization-related interaction type (Table 2), we can infer that these two genes participate in a neutralization-related interaction(s). However, it does not mean that IFNG neutralizes IL12A, or vice versa. We can only say that these two genes interact somehow in a neutralization-related pattern.

It is likely that multiple interaction-related keywords co-exist in one sentence. For example, an IFNG-IL12 neutralization-related sentence is “*In vitro IL-12 neutralization dramatically impaired the IFN-gamma response to S. typhimurium but not to ConA*” [26]. This sentence contains two interaction-related keywords “neutralization” and “impaired”. This is a complex relation where a neutralization of one gene impairs another gene expression. It hints that one gene positively regulates another. In this case, the *neutralization* is really an experimental condition. Our literature mining program retrieved both keywords independently without considering them together. Specifically, our current method identifies all the interaction keywords and maps each of them to corresponding INO interaction terms. However, we have not systematically modeled and integrated these co-existing terms into better understanding of the patterns of corresponding literature text. It would be more advanced if we could process these two keywords simultaneously and assign a unique interaction type, such as ‘impairment after neutralization’, which would be a subclass (or child term) of the existing INO term ‘positive regulation’. While this example demonstrates a new direction of future research, such analysis does not undermine the contributions of the new INO-based literature mining strategy first reported in this manuscript. Indeed, our strategy provides a new start point and platform for further addressing these challenges.

The analysis of vaccine-associated interaction networks requires intensive research. The research reported here uses INO-based literature mining to analyze the vaccine-relevant gene-gene interactions. More research can be conducted to study vaccine-gene interactions and vaccine-associated adverse events. In addition to the PubMed literature resource used in this study, additional public resources such as Semantic MEDLINE, summarizing PubMed results into an interactive graph of semantic predications [27], and The Vaccine Adverse Event Reporting System (VAERS; https://vaers.hhs.gov), collecting vaccine-associated adverse events following the administrations with various licensed vaccines [28], may further improve the INO-based analysis. While Semantic MEDLINE and VAERS have been used in other vaccine-related research [29, 30], INO-based approaches are expected to advance the research on the interaction networks among vaccines, genes, and adverse events. The integrative research combining INO and different resources would further facilitate our understanding of vaccine mechanisms and support public health.

## Conclusions

INO provides a novel approach in ontologically defining hierarchical interaction types and related interaction keywords for literature mining. We have adopted a modified Fisher’s exact test for statistically analyzing the enriched interactions, in terms of INO. The input of such a novel statistical test is the gene-gene interaction pairs together with corresponding INO interaction terms. Such a literature mining strategy was applied and evaluated in the mining of vaccine-associated gene-gene interactions. The results of our study demonstrate that the ontology-based literature mining in combination with an INO-based statistical interaction enrichment test is able to efficiently mine and analyze different types of vaccine-associated gene-gene interactions and corresponding gene pairs.

## Abbreviations

INO: Interaction network ontology
PSI-MI: Proteomics standards initiative-molecular interaction
GO: Gene ontology
VO: Vaccine ontology
BFO: Basic formal ontology
SVM: Support vector machine
CC: Creative commons
NCBO: National center for biomedical ontology
BH: Benjamini-Hochberg
OGG: Ontology of genes and genomes.
